# Occupationally Acquired Polymicrobial Keratitis Caused by Acanthamoeba and Rare Enteric Bacteria Following Gastrointestinal Splash Exposure: A Case Report

**DOI:** 10.1155/crdi/3791851

**Published:** 2026-06-08

**Authors:** Fatma Elnaggar, Heba AlSharif, Muhammad Haris Mazhar, Danya Aldahan, Halah Bin Helayel

**Affiliations:** ^1^ Anterior Segment Division, King Khaled Eye Specialist Hospital, Riyadh, Saudi Arabia, kkesh.med.sa; ^2^ College of Medicine, Alfaisal University, Riyadh, 11533, Saudi Arabia, alfaisal.edu; ^3^ Department of Pharmacy, King Khaled Eye Specialist Hospital, Riyadh, Saudi Arabia, kkesh.med.sa

**Keywords:** acanthamoeba, case report, contact lenses, occupational exposure, polymicrobial keratitis

## Abstract

**Background:**

Polymicrobial keratitis is an uncommon but vision‐threatening condition, particularly when it involves protozoal and bacterial pathogens. Healthcare workers may be at increased risk due to occupational exposure to hospital‐associated microorganisms.

**Case Presentation:**

We report a case of severe polymicrobial keratitis in a 40‐year‐old contact lens‐wearing nurse who developed acute keratitis following inadvertent ocular exposure to nasogastric secretions while caring for a critically ill child. Contact lens culture and in vivo confocal microscopy identified *Acanthamoeba*, *Enterococcus faecalis*, *Staphylococcus capitis*, *and Pantoea* species. The patient developed progressive ring infiltrates, stromal haze, and endothelial dysfunction consistent with advanced infectious keratitis. Intensive antiamoebic and targeted antimicrobial therapy resulted in infection control but with significant corneal scarring requiring planned lamellar keratoplasty.

**Conclusion:**

This case represents a rare example of occupationally acquired polymicrobial keratitis involving Acanthamoeba and multiple healthcare‐associated bacteria. It highlights the potential for gastrointestinal and hospital flora to cause severe ocular infection in contact lens users and underscores the need for heightened infection‐control awareness among healthcare workers.

## 1. Introduction

Polymicrobial keratitis is a rare, sight‐threatening condition accounting for approximately 2%–15% of all infectious keratitis cases [1, 2]. It describes corneal infection caused by two or more microorganisms, including bacteria, viruses, fungi, and protozoa, either from the same or different categories. The severity of the corneal infection depends on the virulence of the causative microbes and the underlying host risk factors [3].

Isolating the infecting organisms is crucial to tailor specific antibiotics, thereby minimizing toxicity and preventing the emergence of antimicrobial resistance [4]. Ophthalmic antibiotics constitute the first‐line treatment for polymicrobial keratitis [5]. In cases refractory to topical therapy, intrastromal antibiotics may be considered, particularly before proceeding to penetrating keratoplasty [6]. Early penetrating keratoplasty may also be indicated in cases presenting with large corneal ulcers [5]. Polymicrobial keratitis carries a significantly poorer visual prognosis compared to monomicrobial infections, largely due to synergistic pathogenicity, as well as the diagnostic and therapeutic challenges [1–3, 7]. Clinicians should, therefore, maintain a high index of suspicion for polymicrobial keratitis in high‐risk patients who fail to respond to initial treatment for infectious keratitis.

Contact lens (CL) wear is the primary risk factor for polymicrobial and monomicrobial keratitis [1, 2]. Factors such as suboptimal CL hygiene, overnight wear, and the type of CL and cleaning solution increase the predisposition to CL‐related keratitis. Following CL wear, ocular trauma is the second most predisposing factor, particularly for polymicrobial keratitis [1, 2]. Additional reported risk factors include topical corticosteroid use, prior corneal surgery, and diabetes mellitus [8]. Jay Hay et al. [9] discussed the increased risk of microbial keratitis among hospital staff who wear CLs, attributed to greater exposure to potentially pathogenic microbes. Similarly, another study reported cases of *Pseudomonas* keratitis among healthcare staff, raising concerns regarding a possible occupational hazard [10]. Herein, we report a case of polymicrobial keratitis caused by *Acanthamoeba, Enterococcus faecalis, Staphylococcus capitis, and Pantoea* species in a CL‐wearing nurse.

## 2. Case Presentation

A 40‐year‐old female nurse presented to the emergency department with a 3‐day history of photophobia, redness, and decreased vision in her right eye after wearing CL overnight. Two days prior to presentation, she had been started on topical moxifloxacin 0.5% (Vigamox) hourly at another hospital, with partial improvement in photophobia but persistent redness and pain. The patient used daily‐wear CL for refractive correction. She reported occasionally sleeping with her lenses but denied any history of ocular trauma, prior ocular infections, showering with lenses, or rinsing them with tap water. The night before symptom onset, she experienced a splash exposure while removing a nasogastric tube, after which she rinsed her eyes with tap water and continued using the same CLs.

**FIGURE 1:**
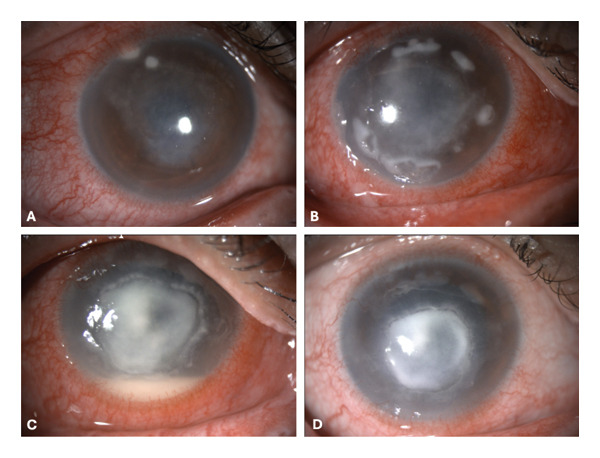
Slit‐lamp photography of the patient’s right eye. (A) Focal discrete corneal infiltrates at 11 o’clock position, measuring 1 × 1 mm at the limbus and 0.5 × 0.5 mm peripherally and a central corneal haze of 6.0 × 4.6 mm. (B) Severe keratitis with 360‐degree peripheral infiltrates and a central ring opacity. (C) Disappearing peripheral infiltrates after 1 week of antimicrobial therapy, denser active central infiltrate, and development of 2‐mm hypopyon. (D) 21 days after starting antiacanthamoeba therapy, showing residual peripheral infiltrates and a stable central deep corneal lesion that started to scar.

Upon examination, her best‐corrected visual acuity (BCVA) was 20/200 OD and 20/20 OS. Right eye slit‐lamp examination revealed moderate ciliary injection, focal discrete white corneal infiltrates measuring 1 × 1 mm at the limbus and 0.5 × 0.5 mm peripherally, a central corneal stromal haze of 6.0 × 4.6 mm in diameter, and an overlying corneal epithelial defect (Figure 1A). The anterior chamber showed a trace of pigmented cells but no hypopyon. Examination of the left eye was unremarkable. A corneal scraping was performed, and the specimen was sent for Gram stain and culture. The patient was started on fortified cefazolin 5% eye drops hourly, fortified ceftazidime 5% eye drops hourly, and cyclopentolate 1% eye drops twice daily. She was advised to return to the emergency room if there were worsening of her symptoms, lack of improvement, or development of endophthalmitis symptoms.

Two days later, the patient presented with persistent symptoms and signs of worsening keratitis. Slit‐lamp examination revealed severe keratitis with 360‐degree ring infiltrates in the peripheral cornea (Figure 1B). Central corneal edema and Descemet’s membrane folds were also observed. The culture of the initial corneal scrape showed growth of *Staphylococcus capiti*s, a gram‐positive, coagulase‐negative bacterium. Sensitivity results showed that it was susceptible to clindamycin, moxifloxacin, and penicillin and resistant to erythromycin. Subsequently, the patient was admitted because of clinical worsening. Fortified antibiotics were temporarily withheld for 12 h to allow for repeat corneal scraping, while lubricants and cycloplegics were maintained. This approach was in accordance with institutional protocol, where suspected infectious keratitis is initially treated as bacterial until proven otherwise. Antibiotics were temporarily withheld prior to repeat sampling to increase microbial yield and reduce the potential inhibitory effect of antibiotics on culture results. A repeat corneal scraping was performed after 12 h of discontinuing antibiotics, and samples were sent for staining, culture, and antibiotic susceptibility. CLs were also sent for microbiological studies.

On the second day of admission, in vivo confocal microscopy was performed, and the findings strongly suggested Acanthamoeba keratitis. A diagnosis of Acanthamoeba keratitis was confirmed, and anti‐Acanthamoeba therapy was added to the treatment regimen using chlorhexidine 0.02% and voriconazole 1%, alternating every hour while awake. Chlorhexidine was selected as the primary biguanide agent in accordance with institutional protocol and local availability, with supporting evidence for the efficacy of voriconazole‐containing regimens in *Acanthamoeba* keratitis. Additionally, topical cyclopentolate was initially increased to three times daily; however, due to persistent inflammation and inadequate pain control, cyclopentolate was discontinued and replaced with atropine 1% twice daily to provide longer acting cycloplegia and improved pain control. After 1 week of treatment, the central infiltrate became sharply demarcated with the development of a 2‐mm hypopyon (Figure 1C).

**FIGURE 2:**
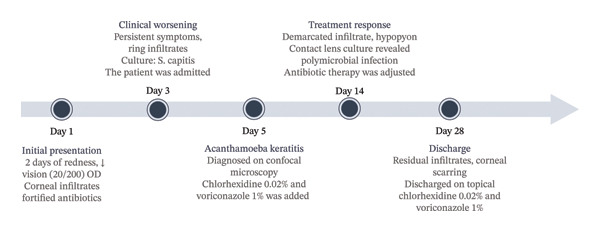
Clinical timeline from initial presentation through hospital discharge.

CL cultures were positive for *Enterococcus faecalis, Staphylococcus capitis, Pantoea* species, *and Acanthamoeba*. According to culture susceptibility results, fortified cefazolin was replaced with vancomycin 2.5% every 6 h. The symptoms improved over the weeks, and the antibiotics were tapered over the admission period. After 21 days of anti‐acanthamoeba treatment, there were minimal residual peripheral ring infiltrates and a central deep infiltrate that started to scar (Figure 1D). The patient was discharged on topical chlorhexidine 0.02% and voriconazole 1% and counseled regarding the necessity of lamellar keratoplasty for visual rehabilitation after corneal quiescence. After keratoplasty, she achieved a final uncorrected visual acuity of 20/160. Figure 2 presents a timeline summarizing key events from the patient’s initial presentation through hospital discharge.

## 3. Discussion

Polymicrobial keratitis is a vision‐threatening corneal infection that poses significant therapeutic challenges. To our knowledge, this is among the first reported cases of *Acanthamoeba* keratitis coexisting with bacterial species that are rarely implicated in corneal infections. The patient’s overnight CL use and occupational exposure to nasogastric secretions were identified as the likely major contributing factors that compromised the corneal epithelium. Given that pathogens can access the ocular surface through aerosolization and that all isolated bacteria are commonly associated with hospital‐acquired infections, we propose that the concurrent infection was acquired from the hospital setting [9, 10].

*Acanthamoeba*, a free‐living protozoan commonly found in soil, water, dust, and air, is a rare cause of CL‐related keratitis, often associated with prolonged treatment courses and poor visual outcomes [1]. While several cases of *Acanthamoeba* keratitis mixed with bacteria and fungi have been reported, the precise role of bacterial co‐infection in the pathogenesis of *Acanthamoeba* keratitis remains unclear [11, 12]. Emerging evidence suggests that *Acanthamoeba* can act not only as a phagocytic predator but also as a host for intracellular microorganisms that evade digestion and persist within the amoeba cell cytoplasm [13]. These intracellular bacteria may survive and replicate, possibly increasing the severity of *Acanthamoeba* keratitis [14].

However, the bacterial isolates identified in our case are not commonly reported as *Acanthamoeba* endosymbionts and are not part of the ocular microbiome [14, 15]. Therefore, they were more likely acquired from the hospital environment, given their known prevalence in such settings and the patient’s recent exposure to biological fluids. We hypothesize that these bacteria created a favorable environment that facilitated *Acanthamoeba* growth rather than representing true endosymbiosis.

*Enterococcus faecalis* is a Gram‐positive commensal bacterium that inhabits the human gastrointestinal canal. It lacks major virulence factors, such as endotoxin and exotoxin production, and typically causes opportunistic infections by adhering to susceptible surfaces [16]. *Enterococcus faecalis* is a major cause of nosocomial urinary tract and bloodstream infections. However, it is rarely implicated in corneal infections and is typically observed as a cause of postoperative endophthalmitis [16, 17]. Isolated cases of enterococcal keratitis and conjunctivitis have also been reported [16, 18].

On the other hand, *Pantoea* species are gram‐negative bacilli belonging to the Enterobacteriaceae family. They often cause hospital‐acquired bloodstream infections, septic arthritis, osteomyelitis, and wound infection in immunocompromised individuals [19]. Unlike other Gram‐negative rods, which are commonly implicated in ocular infections with poor visual outcomes, most reported cases of *Pantoea* keratitis demonstrate susceptibility to standard antibiotics and are associated with favorable visual prognoses [19, 20].

While a relatively low‐virulence organism, *Staphylococcus capitis* is a common cause of morbidity in hospitalized infants. Its main virulence mechanism is biofilm production, which facilitates its adherence [21]. It has been reported as a causative agent of postoperative and traumatic polymicrobial endophthalmitis [22]. Several studies have investigated the reservoirs of *Staphylococcus capitis* within hospital settings, identifying isolates in the nasal cavities of nursing and medical personnel, incubators in neonatal intensive care units, and a range of medical and surgical equipment [21, 23]. Our patient was a nurse who reported inadvertent exposure to secretions during the removal of a nasogastric tube from a child in the intensive care unit.

Overnight CL wear is a well‐established and significant risk factor for infectious keratitis. Studies show that the risk of keratitis is substantially higher in individuals who wear lenses overnight compared to those who use daily disposable lenses [24]. However, the polymicrobial nature of the infection and the involvement of healthcare‐associated pathogens suggest hospital acquisition. Continued CL use in the setting of contamination further compromised corneal defense mechanisms, creating a favorable environment for microbial colonization and growth.

## 4. Conclusion

Polymicrobial keratitis in this case was likely acquired from the hospital environment. The presence of well‐known hospital‐acquired pathogens among the causative organisms indicates a potential occupational risk. Additionally, overnight CL wear probably acted as a reservoir for these microbes, increasing their contact with the corneal surface. Greater emphasis should be placed on educating healthcare professionals regarding CL hygiene and occupational hazards, particularly following ocular exposure events.

## Funding

This study was supported by King Khaled Eye Specialist Hospital.

## Ethics Statement

This research was conducted ethically in accordance with the World Medical Association Declaration of Helsinki. The study protocol was approved by the institutional review board in King Khaled Eye Specialist Hospital under reference no. RD/26001/IRB/0733‐24.

## Consent

Written informed consent was obtained from the patient, authorizing publication of this case report and accompanying images.

## Conflicts of Interest

The authors declare no conflicts of interest.

## Data Availability

Data sharing is not applicable to this article as no datasets were generated or analyzed during the current study.
